# Impact of Sodium-Glucose Cotransporter-2 Inhibitors on Post-Transurethral Resection of Bladder Tumor Infection and Prognosis

**DOI:** 10.3390/diagnostics15141824

**Published:** 2025-07-20

**Authors:** Nobutaka Nishimura, Makito Miyake, Tatsuki Miyamoto, Daiki Ichii, Makito Naoi, Kosuke Narita, Mikiko Kohashi, Atsushi Tomioka, Kazumasa Torimoto, Ryotaro Kawashima, Kazuki Miyazaki, Tomoharu Iwao, Kuniaki Inoue, Toshihiko Matsubara, Kiyohide Fujimoto

**Affiliations:** 1 Department of Urology, Nara Medical University, Nara 634-8522, Japan; nobunishimura@naramed-u.ac.jp (N.N.); kiyokun@naramed-u.ac.jp (K.F.); 2 Department of Urology, Kouseikai Takai Hospital, Tenri 632-0006, Japan; tatsuki8770@gmail.com; 3 Department of Urology, Tane General Hospital, Osaka 550-0025, Japan; aiki0626nmu@gmail.com; 4 Department of Urology, Okanami General Hospital, Iga 518-0121, Japan; makinaoi@yahoo.co.jp; 5 Department of Urology, Matsusaka Chuo General Hospital, Matsusaka 515-0818, Japan; naritacbt48@gmail.com; 6 Department of Urology, Nara City Hospital, Nara 630-8305, Japan; mikiko.onishi.0123@gmail.com; 7 Department of Urology, Saiseikai Chuwa Hospital, Sakurai 633-0054, Japan; tomioka515@yahoo.co.jp; 8 Department of Urology, Nara Prefecture General Medical Center, Nara 630-8054, Japan; torimoto@nara-hp.jp; 9 Department of Urology, Osaka Kaisei Hospital, Osaka 532-0003, Japan; kawachan.nara.bsk.13.6.13@gmail.com; 10 Department of Urology, Hoshigaoka Medical Center, Hirakata 573-0013, Japan; haruwoaisuruhito815@gmail.com; 11 Department of Urology, Hirao Hospital, Nara 634-0076, Japan; hinnyoutomoharu@outlook.jp; 12 Department of Urology, Osaka Gyoumeikan Hospital, Osaka 554-0012, Japan; kuniaki1234@icloud.com; 13 Department of Urology, Nara Prefecture Seiwa Medical Center, Ikoma, Nara 636-0802, Japan; agemomitm@gmail.com

**Keywords:** diabetes mellitus, sodium-glucose cotransporter-2 inhibitor, transurethral resection of bladder cancer, pyuria, urinary tract infection

## Abstract

**Background/Objectives:** Sodium-glucose cotransporter-2 inhibitors (SGLT2is), by elevating urinary glucose levels, may predispose patients to urinary tract infections (UTI). However, limited evidence is available regarding the association between SGLT2is and postoperative outcomes after transurethral resection of bladder tumors (TURBT). We evaluated the impact of SGLT2is on post-TURBT pyuria and febrile UTI (fUTI), as well as oncological outcomes. **Methods:** We retrospectively reviewed the data of 812 patients with and without diabetes mellitus (DM) who underwent TURBT between January 2019 and May 2024. The patients were categorized into three groups: non-DM (Nara Medical University cohort, *n* = 344), DM non-SGLT2i (multi-institutional cohort, *n* = 363), and DM SGLT2i (multi-institutional cohort, *n* = 105). We compared fUTI-free survival, fUTI-related hospitalization-free survival, and persistent pyuria duration as well as oncological outcomes using the inverse probability of treatment weighting (IPTW)-adjusted Kaplan–Meier method and Cox regression analysis. **Results:** No significant differences in fUTI-free or hospitalization-free survival were observed between the non-DM and DM groups, whereas the DM group had prolonged pyuria compared to the non-DM group (1-year pyuria rate: 36.6% vs. 18.2%; *p* < 0.001). In contrast, the DM SGLT2i group had significantly shorter fUTI-free survival (1-year fUTI-free survival: 83.0% vs. 90.0%; *p* = 0.013) and longer pyuria persistence (1-year pyuria rate: 70.6% vs. 28.9%; *p* < 0.001) than the DM non-SGLT2i group. Additionally, the DM SGLT2i group was significantly associated with shorter UTUC-free survival than the DM non-SGLT2i group (*p* = 0.0072). SGLT2i was an independent prognostic factor for fUTI and prolonged pyuria in IPTW-adjusted Cox regression analysis. No significant differences were observed in fUTI-free survival, hospitalization-free survival, or persistent pyuria duration among the different SGLT2i types. **Conclusions:** Temporal discontinuation of SGLT2i in the peri-TURBT period may prevent persistent postoperative pyuria and the risk of fUTI.

## 1. Introduction

After the diagnosis of bladder cancer, most patients undergo transurethral resection of the bladder tumor (TURBT), which is considered one of the least invasive surgical procedures in urology [1]. However, several comorbidities must also be considered [2]. Urinary tract infections (UTIs) are the most prevalent complications of TURBT. The overall complication rate was 4.3%, with UTIs occurring in 3% of the cases and hematuria in 2.1% [3]. Multiple risk factors for UTI have been identified in previous studies, including prior pelvic radiotherapy, tumor size, preoperative hospital stay, and the presence of pyuria and bacteriuria [4], which can lead to prolonged hospitalization or sepsis [5]. Therefore, prevention of febrile urinary tract infections (fUTIs) is essential.

Numerous risk factors for pyuria have been identified. We focused on sodium-glucose cotransporter-2 inhibitors (SGLT2is), a class of medications used to manage type 2 diabetes mellitus (T2DM) as well as heart failure and kidney disease. SGLT2is block the reabsorption of glucose in the kidneys, resulting in increased glucose excretion in the urine and lowered blood glucose levels. Furthermore, the loss of glucose through the urine creates a calorie deficit, which can contribute to weight loss and improve T2DM management [6,7]. Additionally, SGLT2is offer protective benefits for renal function, diabetic nephropathy, and heart failure due to their ability to promote osmotic diuresis, reduce intravascular volume, and lower blood pressure. In addition, SGLT2is decrease intraglomerular pressure and proteinuria, helping to slow the progression of kidney disease [8,9,10]. These mechanisms contribute to improved cardiovascular and renal outcomes in patients receiving SGLT2 inhibitors. Many patients with T2DM are prescribed SGLT2is due to these advantageous mechanisms. Numerous reports have demonstrated the safety of SGLT2i use in older patients with T2DM and in those with kidney or cardiovascular diseases [11,12]. However, the mechanism by which SGLT2is increase urinary glucose levels may also contribute to a higher incidence of UTIs [13,14,15]. In fact, meta-analyses have shown that the use of SGLT2is is associated with an increased risk of UTIs; however, these agents are also linked to improved cardiometabolic and renal outcomes, including a reduced incidence of heart failure and kidney disease events [16,17].

We were concerned about the potential association between SGLT2i use and the increased incidence of fUTI during the perioperative period of TURBT. After transurethral surgery, the urethral or vesical mucosa typically sustains some injury due to endoscope manipulation, which can compromise the barrier function against bacterial infection [18,19]. If SGLT2i use leads to fUTI after TURBT, it may be necessary to discontinue SGLT2i administration during the perioperative period and consider re-initiating treatment only after the resolution of pyuria. In this study, we evaluated the association between SGLT2i use and genitourinary adverse events after TURBT.

## 2. Materials and Methods

### 2.1. Study Design and Patient Selection

The study protocol was approved by the Institutional Review Board for Clinical Studies of Nara Medical University (Medical Ethics Committee ID2891). The study was conducted in accordance with the principles of the Declaration of Helsinki.

This retrospective chart review included patients who underwent TURBT for bladder cancer between January 2019 and May 2024 at the 13 participating institutions. All patients received perioperative antibiotic treatment, with no antibiotics remaining at the time of TURBT. Patients with incomplete data or those with end-stage renal disease (ESRD) who were undergoing hemodialysis were excluded. Initially, the patients were categorized into those with type 1 or 2 DM (DM group) in the multi-institutional cohort and those without DM (non-DM group) in the Nara Medical University cohort. Subsequently, patients in the DM group were further classified into those treated with SGLT2is for T2DM (DM SGLT2i group) and those treated with other diabetes therapies (DM non-SGLT2i group). Functional and oncological outcomes were compared between the DM and non-DM groups and between the DM SGLT2i and DM non-SGLT2i groups.

### 2.2. Outcomes

Primary outcomes included functional prognoses such as fUTI-free survival, fUTI-related hospitalization-free survival, and persistent pyuria duration. fUTI-free survival was defined as the time from the date of TURBT to the first occurrence of fUTI, and fUTI-related hospitalization-free survival was defined as the time from the date of TURBT to hospitalization for fUTI. The persistent pyuria duration was defined as the time from the date of TURBT to the resolution of pyuria, with the date of BCG therapy instillation censored only for this outcome. For patients with muscle-invasive bladder cancer (MIBC), the date of initiation of additional treatments, such as radical cystectomy or radiation therapy, was defined as the censoring point. To examine the specific effects of different SGLT2is on the incidence of fUTI and persistent pyuria, the outcomes were evaluated across various SGLT2i types, including ipragliflozin, dapagliflozin, luseogliflozin, tofogliflozin, canagliflozin, and empagliflozin. Prognostic factors for fUTI incidence and persistent pyuria were analyzed using IPTW-adjusted Cox proportional hazards models.

Secondary outcomes included intravesical recurrence-free survival (IVRFS), progression-free survival (PFS), and upper urinary tract urothelial carcinoma-free survival (UTUC-free survival). In this analysis, patients with pT0 and MIBC were excluded from the IPTW-adjusted population, and oncological outcomes were assessed exclusively in patients with NMIBC.

### 2.3. Statistical Analysis

To minimize selection bias between the DM and non-DM groups, as well as the DM SGLT2i and DM non-SGLT2i groups, inverse probability of treatment weighting (IPTW)-adjusted Kaplan–Meier survival analyses and Cox regression models were applied. Covariates for IPTW adjustment were selected based on previous literature [20,21,22,23,24].

Quantitative variables are expressed as mean (standard deviation [SD]), and categorical variables are expressed as proportions. Patient characteristics were analyzed using standardized mean differences (SMD). Functional and oncological outcomes were evaluated using IPTW-adjusted log-rank tests. For comparison among the sixSGLT2i types, these functional outcomes were assessed using log-rank tests. Survival curves were generated using R (version 4.4.2; R Core Team, 2024 [25]) and RStudio (version 2024.09.0 + 375; RStudio, PBC, 2024). The “survey” package (version 4.4-2) was used to calculate IPTW weights, and weighted Kaplan–Meier survival curves were generated using the “survminer” package (version 0.5.0; function: ggsurvplot). The “survival” package (version 3.8-3) was also utilized for Cox proportional hazards modeling and survival curve estimation. Additional statistical analyses were performed using EZR software (version 1.68; Saitama Medical Center, Jichi Medical University, Saitama, Japan). A *p* value of less than 0.05 was considered statistically significant.

## 3. Results

### 3.1. Patient Selection

**Figure 1:**
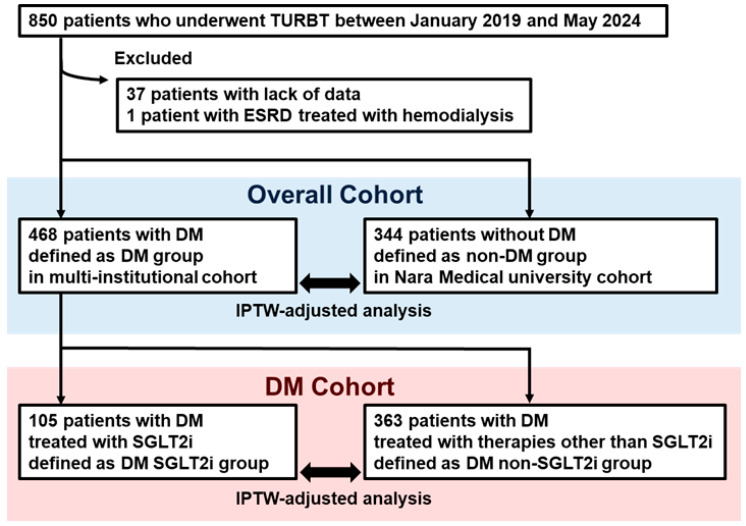
**Flow chart of patient selection.** Patient selection flow chart. Overall cohort included 468 patients with DM in the multi-institutional cohort (DM group) and 344 patients without DM in the Nara Medical University cohort (non-DM group). The DM cohort included 105 patients treated with SGLT2is (DM SGLT2i group) and 363 patients treated with other antidiabetic medications (DM non-SGLT2i group). Functional outcomes after TURBT were compared between the DM and non-DM groups as well as between the DM SGLT2i and DM non-SGLT2i groups. Abbreviations: DM = diabetes mellitus; ESRD = end-stage renal disease; SGLT2i = sodium-glucose cotransporter-2 inhibitor; TURBT = transurethral resection of bladder tumor.

Figure 1 shows a flowchart of the patient selection. Of the 850 patients who underwent TURBT between January 2019 and May 2024, 37 were excluded because of incomplete data and one because of hemodialysis for ESRD, resulting in a final cohort of 812 patients for this analysis. First, within the overall cohort, 468 patients in DM group and 344 patients in the non-DM group were identified. Second, within the DM cohort, 105 patients in the DM SGLT2i group and 363 patients in the DM non-SGLT2i group were identified.

### 3.2. Comparison of Functional and Oncological Outcomes Between DM and Non-DM Groups

Table 1 shows the clinicopathological characteristics of the DM and non-DM groups in both unweighted and IPTW-adjusted populations. Even after IPTW adjustment, substantial differences remained in Eastern Cooperative Oncology Group-performance status (ECOG-PS) and the proportion of patients receiving induction or maintenance Bacille Calmette–Guérin (BCG) therapy (SMD > 0.2).

**Figure 2:**
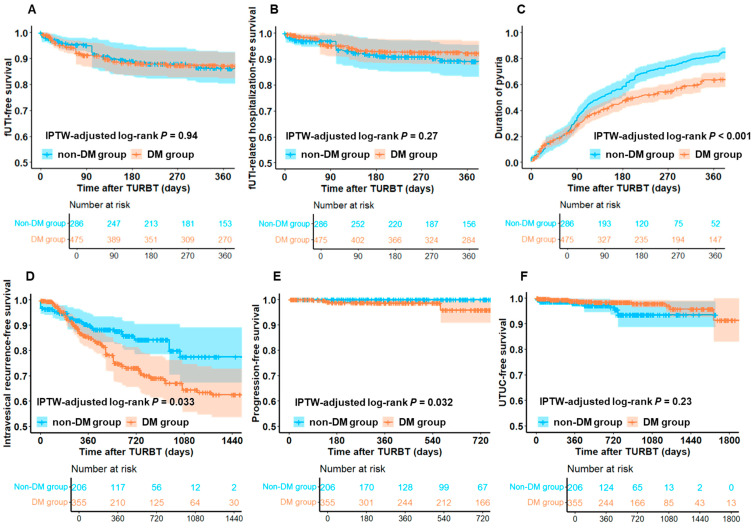
**Functional and oncological outcomes after TURBT in DM and non-DM groups.** This figure shows the comparison of functional outcomes, including fUTI-free survival (**A**), fUTI-related hospitalization-free survival (**B**), and persistent pyuria duration (**C**), and oncological outcomes including IVRFS (**D**), PFS (**E**), and UTUC-free survival (**F**) between the DM and non-DM groups. In both groups, covariates related to the clinicopathological variables were balanced using IPTW adjustment, and survival curves were generated accordingly. IPTW-adjusted Kaplan–Meier curves are depicted with 95% CI areas. Abbreviations: DM = diabetes mellitus; fUTI = febrile urinary tract infection; IPTW = inverse probability of treatment weighting; IVRFS = intravesical recurrence-free survival; PFS = progression-free survival; TURBT = transurethral resection of bladder tumor; UTUC = upper urinary tract urothelial carcinoma.

Figure 2 shows functional and oncological outcomes in the DM and non-DM groups. The DM group was significantly associated with a longer persistent pyuria duration than the non-DM group (Figure 2C). Additionally, the DM group had a significantly shorter IVRFS and PFS compared to the non-DM group (Figure 2D,E).

### 3.3. Comparison of Functional and Oncological Outcomes Between DM SGLT2i and DM Non-SGLT2i Groups

Table 2 shows the clinicopathological characteristics of the DM SGLT2i and DM non-SGLT2i groups in both the unweighted and IPTW-adjusted populations. The SMD for ECOG-PS remained above 0.2 between the two groups even after IPTW adjustment.

**Figure 3:**
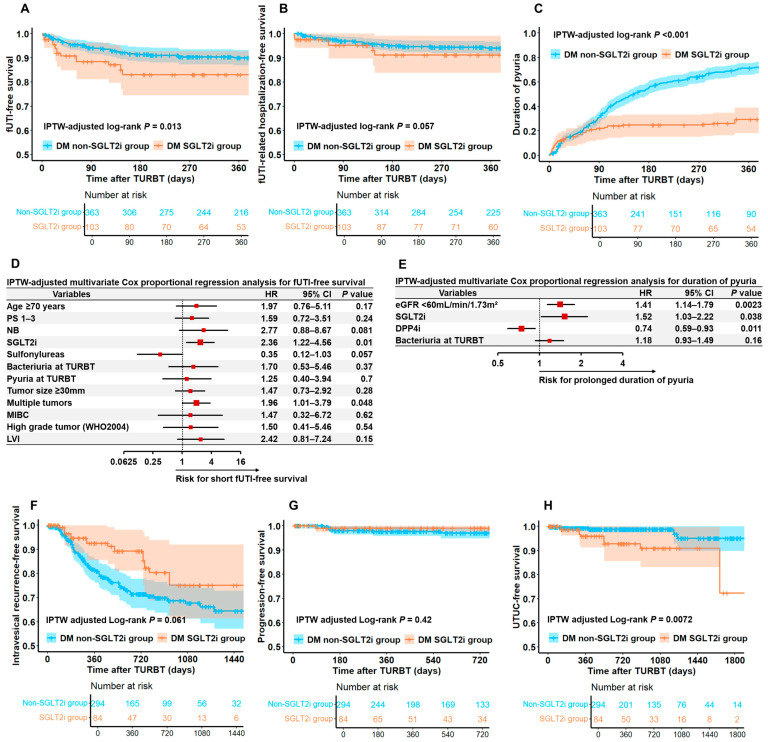
**Functional and oncological outcomes after TURBT in DM SGLT2i and DM non-SGLT2i groups.** This figure shows the comparison of functional outcomes, including fUTI-free survival (**A**), fUTI-related hospitalization-free survival (**B**), and persistent pyuria duration (**C**) between the SGLT2i and non-SGLT2i groups. HRs (95% CI) from IPTW-adjusted multivariate Cox proportional regression analysis for fUTI-free survival (**D**) and duration of pyuria (**E**) are shown as forest plots. Additionally, the comparison of oncological outcomes including IVRFS (**F**), PFS (**G**), and UTUC-free survival (**H**) are also shown between the SGLT2i and non-SGLT2i groups. In both groups, covariates related to the clinicopathological variables were balanced using IPTW adjustment, and survival curves were generated accordingly. IPTW-adjusted Kaplan–Meier curves are depicted with 95% CI areas. Abbreviations: CI = confidence interval; DM = diabetes mellitus; fUTI = febrile urinary tract infection; HR = hazard ratio; IPTW = inverse probability of treatment weighting; IVRFS = intravesical recurrence-free survival; PFS = progression-free survival; TURBT = transurethral resection of bladder tumor; UTUC = upper urinary tract urothelial carcinoma.

Figure 3 shows functional and oncological outcomes in the DM SGLT2i and DM non-SGLT2i groups and the hazard ratio for fUTI and prolonged pyuria from IPTW-adjusted multivariate Cox proportional regression analysis. Use of an SGLT2i was significantly associated with shorter fUTI-free survival and longer persistent pyuria duration than the use of other antidiabetic drugs. In the IPTW-adjusted multivariate Cox proportional regression analysis, the use of an SGLT2i was a significant independent risk factor for short fUTI-free survival and persistent pyuria (hazard ratio [HR] 2.36; 95% confidence interval [95% CI], 1.22−4.56; *p* = 0.01 and HR 1.52; 95% CI, 1.03−2.22; *p* = 0.038, respectively) (Figure 3D,E). Additionally, the use of an SGLT2i was significantly associated with a higher incidence of UTUC after TURBT (Figure 3H). Supplementary Tables S1 and S2 show the more detailed HR for fUTI-free survival and persistent pyuria based on univariate and multivariate IPTW-adjusted Cox proportional hazards models.

### 3.4. Functional Outcomes of Differences in Each Type of SGLT2i

**Figure 4:**
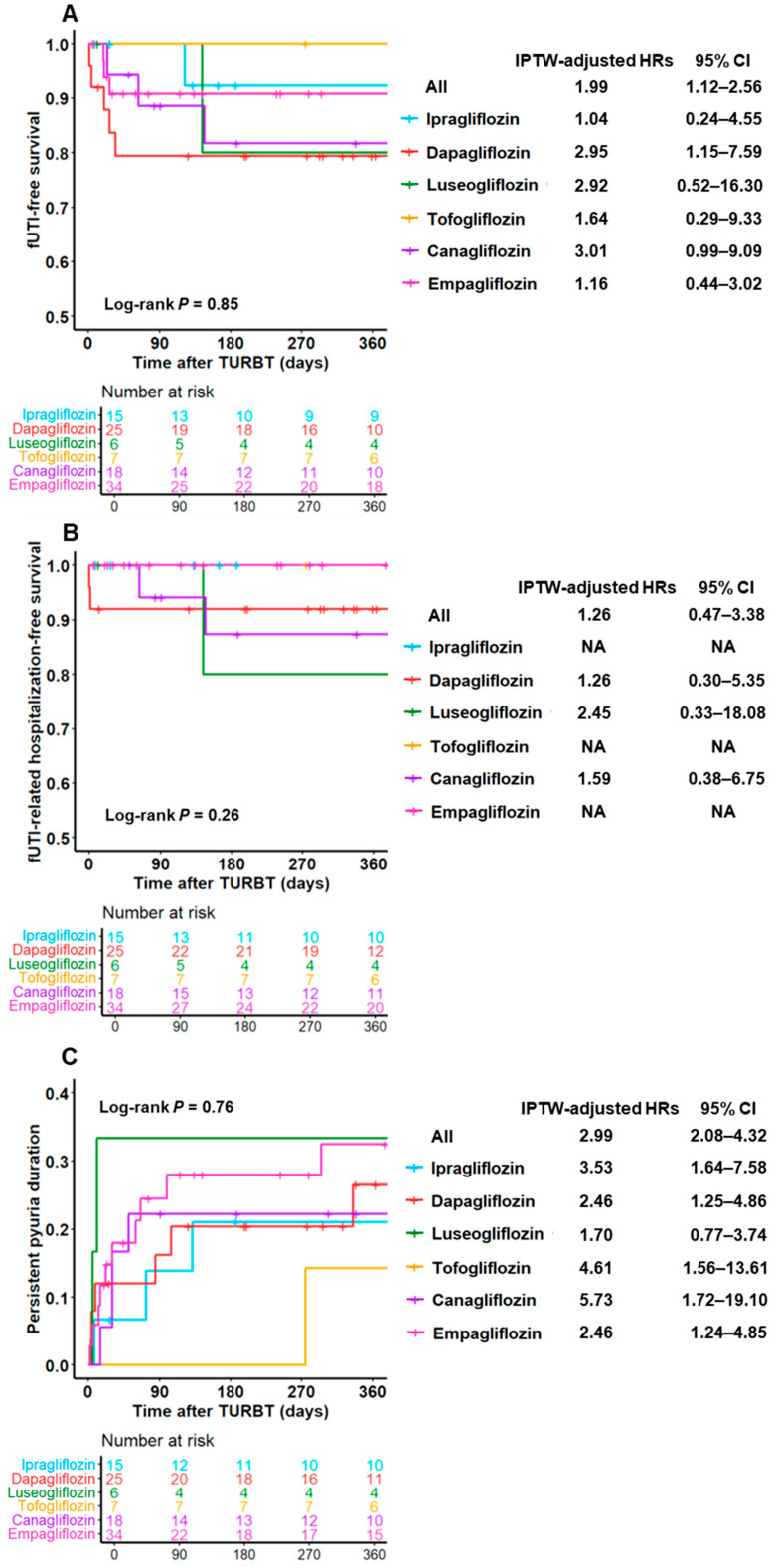
**Functional outcomes after TURBT in each type of SGLT2i.** Functional outcomes, including fUTI-free survival (**A**), fUTI-related hospitalization-free survival (**B**), and persistent pyuria duration (**C**), compared across the six SGLT2 types, including ipragliflozin, dapagliflozin, luseogliflozin, tofogliflozin, canagliflozin, and empagliflozin. HRs and 95% CIs were calculated and compared with those in the DM non-SGLT2i group. Abbreviations: DM = diabetes mellitus; fUTI = febrile urinary tract infection; IPTW = inverse probability of treatment weighting; NA = not applicable; TURBT = transurethral resection of bladder tumor.

Figure 4 shows fUTI-free survival, fUTI-related hospitalization-free survival, and persistent pyuria duration among the six SGLT2i types. The HRs and 95% CIs shown in Figure 4 were calculated and compared with the DM non-SGLT2i group. No significant differences in these functional outcomes were observed among these types. However, dapagliflozin demonstrated a tendency toward shorter fUTI-free survival and longer persistent pyuria duration than other antidiabetic drugs. Among the six SGLT2i types, only dapagliflozin was significantly associated with shorter fUTI-free survival.

## 4. Discussion

SGLT2is are known to increase urinary glucose levels due to their mechanism of action, which involves inhibiting glucose reabsorption in the proximal renal tubules. This results in elevated glucose concentrations in the urine. However, whether this increase directly contributes to a higher incidence of UTI or fUTI remains controversial [26,27]. We highlight that patients who undergo TURBT are inherently at higher risk for severe UTIs, as the procedure causes injury to the urethral and vesical mucosa, thereby disrupting the natural mucosal barrier against uropathogenic bacteria [18,19]. Consequently, this population is considered to be at elevated risk for post-operative UTIs.

Based on this background, we hypothesized that the use of an SGLT2i would further increase the risk of UTI in post-TURBT patients compared to those not receiving SGLT2i therapy. In this context, our study demonstrated that SGLT2i use was associated with a higher incidence of fUTI and a prolonged duration of persistent pyuria following TURBT. These findings suggest that temporarily discontinuing SGLT2i use prior to surgery may help prevent severe fUTI-related complications in this high-risk population.

We also assessed the association between each SGLT2i type and the incidence of fUTI or persistent pyuria (Figure 4). Canagliflozin and dapagliflozin showed a tendency toward higher fUTI incidence, fUTI-related hospitalizations, and prolonged pyuria, whereas empagliflozin appeared less likely to cause these outcomes. In vivo experiments showed that dapagliflozin and canagliflozin were associated with more sustained urinary glucose levels than tofogliflozin, which is consistent with our findings regarding the duration of persistent pyuria [28]. These differences in outcomes observed between these products may be related to the duration of action of the drugs, which can be divided into two categories: long-acting (ipragliflozin and dapagliflozin) and intermediate-acting (tofogliflozin, canagliflozin, empagliflozin, and luseogliflozin) [29]. Given the tendency of dapagliflozin to increase the incidence of fUTI compared to other SGLT2is, we propose that long-acting SGLT2is, such as ipragliflozin and dapagliflozin, may require an extended discontinuation period around the TURBT procedure to minimize these risks.

Figure 2D,E show the significantly shorter IVRFS and PFS observed in patients with DM compared to those without DM. This finding aligns with those of previous studies that have shown DM’s negative impact on NMIBC recurrence and progression, supporting a well-established consensus [30]. Additionally, Figure 3H shows that SGLT2i use was significantly associated with a poorer prognosis of UTUC-free survival than non-use; therefore, our findings introduce a novel factor that could influence post-TURBT management strategies [31]. We hypothesized that the chronic pro-inflammatory effects associated with SGLT2i use may promote the progression or clinical manifestation of precancerous lesions of UTUC in patients with NMIBC, leading to their detection. Although it remains unclear whether mitigating persistent pyuria can reduce UTUC recurrence, discontinuing SGLT2i use post-TURBT could potentially improve oncological and functional outcomes.

Figure 3D,E indicate that an SU or DPP4i may improve functional outcomes after TURBT, whereas SGLT2i use was associated with poorer prognoses. Specifically, the non-use of SU and DPP4i was significantly associated with shorter fUTI-free survival and prolonged pyuria duration, respectively. These drugs have substantial evidence supporting their efficacy in lowering blood glucose levels without affecting urinary glucose levels, in contrast to SGLT2is. Given this difference, it may be beneficial to temporarily switch patients from SGLT2is to an SU or DPP4i during the perioperative period of TURBT. To establish novel strategies for the perioperative period of TURBT, further prospective trials are necessary to compare the functional outcomes between SGLT2is and other antidiabetic agents.

This study has some limitations. First, as this was a retrospective study, it was susceptible to selection bias. Although we applied the IPTW adjustment to minimize bias, not all confounding variables were fully accounted for. In particular, imbalances in patient characteristics—such as ECOG performance status and variations in BCG therapy information—may have influenced the oncological outcomes, potentially introducing unintentional bias in this study. Second, when calculating fUTI-free and fUTI-related hospitalization-free survival, we could not entirely exclude the impact of invasive treatments such as a second TUR and BCG therapy. Although univariate Cox proportional analysis was used to evaluate significant variables, and multivariate analysis was adjusted for these factors, the IPTW-adjusted analysis balanced but did not entirely exclude these impacts. Third, the use of antibiotic agents was not standardized, as clinical pathways and TURBT regimens varied among institutions. This is an inherent limitation of multi-institutional retrospective analyses. We must also emphasize that variations in antibiotic usage may introduce unintentional bias and could have affected our results. Fourth, information regarding preoperative urinalysis, urine culture, and the management of asymptomatic pyuria or bacteriuria prior to TURBT was not consistently available across all participating institutions. As a result, we could not include these factors as covariates in our analysis, which may have introduced unmeasured confounding. Additionally, data on the use of medications for BPH or OAB were incomplete and could not be reliably included as covariates in the IPTW-adjusted analysis. Although we attempted to account for urinary management, including bladder catheterization, the lack of comprehensive data on BPH/OAB medication use remains a limitation that may have influenced our results.

## 5. Conclusions

Our study demonstrated that SGLT2i use is associated with an increased incidence of fUTI, prolonged pyuria duration, and UTUC recurrence after TURBT. SGLT2i use was identified as a significant risk factor for poor functional and oncological outcomes in this setting. However, these findings are based on retrospective observational data, and causality cannot be established. Therefore, further prospective studies, ideally randomized controlled trials, are warranted to confirm these associations and to evaluate the safety and effectiveness of temporarily discontinuing SGLT2i use or switching to alternative antidiabetic agents during the perioperative period.

## Figures and Tables

**Table 1:** Comparison of clinicopathological characteristics of the DM and non-DM groups in both unweighted and IPTW-adjusted populations.

Variables	Unweighted Population			IPTW-Adjusted Population		
	DM Group	Non-DM Group	SMD	DM Group	Non-DM Group	SMD
	*n* (%)	*n* (%)		%	%	
N	468	344		475	286	
Age, years, mean ± SD	75.8 ± 8.6	71.8 ± 11.2	0.40	74.8	73.4	0.15
Sex			0.08			0.01
Male	406 (86.8%)	289 (84.0%)		85.3%	85.6%	
Female	62 (13.2%)	55 (16.0%)		14.7%	14.4%	
ECOG-PS			0.42			0.22
0	373 (79.7%)	277 (80.5%)		78.9%	77.4%	
1	63 (13.5%)	29 (8.4%)		11.7%	15.5%	
2	21 (4.5%)	7 (2.0%)		4.3%	1.6%	
3	4 (0.9%)	0 (0.0%)		0.5%	0.0%	
Unknown	7 (1.5%)	31 (9.0%)		4.6%	5.4%	
Smoking history			0.51			0.12
Never	145 (31.0%)	53 (15.4%)		23.7%	27.4%	
Former	171 (36.5%)	133 (38.7%)		39.4%	38.2%	
Current	76 (16.2%)	42 (12.2%)		14.3%	11.2%	
Unknown	76 (16.2%)	116 (33.7%)		22.5%	23.2%	
BMI, kg/m^2^, mean ± SD	23.5 ± 3.9	23.2 ± 3.5	0.09	23.4	23.7	0.08
Urinary management			0.19			0.14
Normal	460 (98.3%)	344 (100.0%)		99.0%	100.0%	
Self-catheterization	3 (0.6%)	0 (0.0%)		0.4%	0.0%	
Urinary catheter placement	5 (1.1%)	0 (0.0%)		0.6%	0.0%	
Comorbidity						
Heart disease	131 (28.0%)	27 (7.8%)	0.54	19.9%	13.5%	0.17
BPH	126 (26.9%)	90 (26.2%)	0.02	25.3%	21.4%	0.09
NB	16 (3.4%)	7 (2.0%)	0.09	2.4%	1.8%	0.04
OAB	36 (7.7%)	13 (3.8%)	0.17	6.2%	4.2%	0.09
History of pelvic radiation therapy			0.20			0.04
Yes	15 (3.2%)	27 (7.8%)		5.8%	4.8%	
No	453 (96.8%)	317 (92.2%)		94.2%	95.2%	
Bacteriuria at TURBT			0.26			0.20
Yes	112 (23.9%)	123 (35.8%)		28.3%	37.5%	
No	356 (76.1%)	221 (64.2%)		71.7%	62.5%	
Pyuria at TURBT			0.28			0.01
Yes	159 (34.0%)	163 (47.4%)		38.8%	38.3%	
No	309 (66.0%)	181 (52.6%)		61.2%	61.7%	
History of recurrence			0.13			0.08
Primary	357 (76.3%)	280 (81.4%)		78.3%	74.9%	
Recurrence	111 (23.7%)	64 (18.6%)		21.7%	25.1%	
Tumor size			0.09			0.03
<30 mm	384 (82.1%)	271 (78.8%)		81.8%	81.9%	
≥30 mm	73 (15.6%)	62 (18.0%)		15.3%	15.6%	
Undefined	11 (2.4%)	11 (3.2%)		2.9%	2.5%	
Multiplicity			0.22			0.08
Single	234 (50.0%)	186 (54.1%)		51.0%	53.7%	
Multiple	212 (45.3%)	127 (36.9%)		41.3%	40.3%	
Undefined	22 (4.7%)	31 (9.0%)		7.7%	6.0%	
Pathological T stage			0.33			0.14
T0	36 (7.7%)	53 (15.4%)		11.6%	11.7%	
Ta	235 (50.2%)	131 (38.0%)		44.7%	49.7%	
Tis	43 (9.2%)	28 (8.1%)		10.0%	7.3%	
T1	101 (21.6%)	74 (21.5%)		20.6%	17.3%	
MIBC	53 (11.3%)	58 (16.9%)		13.2%	14.0%	
Grade (WHO2004)			0.25			0.16
Low grade	190 (40.6%)	119 (34.6%)		38.0%	45.5%	
High grade	242 (51.7%)	172 (50.0%)		50.6%	43.0%	
No malignancy	36 (7.7%)	53 (15.4%)		11.4%	11.5%	
Concomitant CIS			0.04			0.05
Yes	63 (13.5%)	42 (12.2%)		12.4%	10.9%	
No	405 (86.5%)	302 (87.8%)		87.6%	80.1%	
LVI			0.12			0.04
Yes	34 (7.3%)	37 (10.8%)		8.7%	7.6%	
No	434 (92.7%)	307 (89.2%)		91.3%	92.4%	
Second TUR			0.10			0.05
Yes	62 (13.2%)	58 (16.9%)		13.6%	12.0%	
No	406 (86.8%)	286 (83.1%)		86.4%	88.0%	
Induction BCG therapy			0.62			0.25
Yes	76 (16.2%)	34 (9.9%)		9.3%	5.5%	
No	392 (83.8%)	310 (90.1%)		90.7%	94.5%	
Maintenance BCG therapy			0.29			0.21
Yes	18 (3.8%)	0 (0.0%)		2.2%	0.0%	
No	450 (96.2%)	344 (100.0%)		97.8%	100.0%	

BCG, Bacillus Calmette–Guérin; BMI, body mass index; BPH, benign prostatic hyperplasia; CIS, carcinoma in situ; ECOG-PS, Eastern Cooperative Oncology Group Performance Status; LVI, lymphovascular invasion; MIBC, muscle-invasive bladder cancer; NB, neurogenic bladder; OAB, overactive bladder; TURBT, transurethral resection of bladder tumor; SD, standard deviation; SMD, standardized mean difference; WHO, World Health Organization.

**Table 2:** Comparison of clinicopathological characteristics of the DM SGLT2i and DM non-SGLT2i groups in both the unweighted and IPTW-adjusted populations.

Variables	Unweighted Population			IPTW-Adjusted Population		
	DM SGLT2i Group	DM Non-SGLT2i Group	SMD	DM SGLT2i Group	DM Non-SGLT2i Group	SMD
	*n* (%)	*n* (%)		%	%	
N	105	363		103	363	
Age, years, mean ± SD	74.3 ± 8.5	76.2 ± 8.6	0.22	76.2	75.8	0.05
Sex			0.11			0.19
Male	94 (89.5%)	312 (86.0%)		81.5%	86.5%	
Female	11 (10.5%)	51 (14.0%)		18.5%	13.5%	
ECOG-PS			0.23			0.23
0	87 (82.9%)	286 (78.8%)		75.0%	79.3%	
1	14 (13.3%)	49 (13.5%)		19.2%	13.7%	
2	3 (2.9%)	18 (5.0%)		5.4%	4.9%	
3	1 (1.0%)	3 (0.8%)		0.4%	0.8%	
Unknown	0 (0.0%)	7 (1.9%)		0.0%	1.3%	
Smoking history			0.12			0.08
Never	29 (27.6%)	116 (32.0%)		30.8%	30.3%	
Former	42 (40.0%)	129 (35.5%)		39.8%	37.5%	
Current	18 (17.1%)	58 (16.0%)		14.0%	16.4%	
Unknown	16 (15.2%)	60 (16.5%)		15.5%	15.9%	
BMI, kg/m^2^, mean ± SD	23.8 ± 3.8	23.5 ± 3.9	0.07	23.5	23.6	0.00
Urinary management			0.21			0.19
Normal	105 (100.0%)	355 (97.8%)		100.0%	98.2%	
Self-catheterization	0 (0.0%)	3 (0.8%)		0.0%	0.7%	
Urinary catheter placement	0 (0.0%)	5 (1.4%)		0.0%	1.1%	
Comorbidity						
Heart disease	34 (32.4%)	97 (26.7%)	0.12	22.8%	27.0%	0.10
BPH	28 (26.7%)	98 (27.0%)	0.01	22.4%	26.8%	0.10
NB	3 (2.9%)	13 (3.6%)	0.04	1.4%	2.7%	0.09
OAB	7 (6.7%)	29 (8.0%)	0.05	4.9%	7.4%	0.11
History of pelvic radiation therapy			0.03			0.09
Yes	3 (2.9%)	12 (3.3%)		1.6%	2.9%	
No	102 (97.1%)	351 (96.7%)		98.4%	97.1%	
Blood examination at TURBT						
eGFR, mL/min/1.73 m^2^, mean ± SD	56.7 ± 15.6	58.4 ± 17.8	0.11	57.7	58.1	0.03
HbA1c, %, mean ± SD	7.2 ± 0.8	7.2 ± 1.1	0.03	7.23	7.24	0.01
Antidiabetic drugs						
Insulin	9 (8.6%)	38 (10.5%)	0.07	8.0%	9.6%	0.06
Sulfonylureas	19 (18.1%)	81 (22.3%)	0.11	23.7%	22.1%	0.04
Biguanides	35 (33.3%)	85 (23.4%)	0.22	29.9%	25.3%	0.10
DPP4i	51 (48.6%)	234 (64.5%)	0.33	62.4%	61.6%	0.02
Thiazolidinediones	6 (5.7%)	21 (5.8%)	0.00	3.3%	5.4%	0.11
GLP-1 receptor agonists	1 (1.0%)	4 (1.1%)	0.02	4.0%	10.0%	0.07
α-glucosidase inhibitors	11 (10.5%)	30 (8.3%)	0.08	12.0%	8.9%	0.10
Meglitinides	4 (3.8%)	10 (2.8%)	0.06	2.2%	2.9%	0.05
Bacteriuria at TURBT			0.05			0.02
Yes	27 (25.7%)	85 (23.4%)		22.0%	22.9%	
No	78 (74.3%)	278 (76.6%)		78.0%	77.1%	
Pyuria at TURBT			0.07			0.02
Yes	33 (31.4%)	126 (34.7%)		33.5%	32.6%	
No	72 (68.6%)	237 (65.3%)		66.5%	67.4%	
History of recurrence			0.31			0.02
Primary	90 (85.7%)	267 (73.6%)		76.5%	75.5%	
Recurrence	15 (14.3%)	96 (26.4%)		23.5%	24.5%	
Tumor size			0.11			0.05
<30 mm	85 (81.0%)	299 (82.4%)		84.3%	82.8%	
≥30 mm	16 (15.2%)	57 (15.7%)		14.2%	15.1%	
Undefined	4 (3.8%)	7 (1.9%)		1.5%	2.1%	
Multiplicity			0.21			0.03
Single	52 (49.5%)	182 (50.1%)		48.8%	50.1%	
Multiple	44 (41.9%)	168 (46.3%)		46.5%	45.1%	
Undefined	9 (8.6%)	13 (3.6%)		4.8%	4.8%	
Pathological T stage			0.25			0.13
T0	8 (7.6%)	29 (8.0%)		6.3%	7.5%	
Ta	51 (48.6%)	183 (50.4%)		55.8%	51.7%	
Tis	11 (10.5%)	32 (8.8%)		6.0%	8.9%	
T1	28 (26.7%)	73 (20.1%)		21.4%	20.9%	
MIBC	7 (6.7%)	46 (12.7%)		10.6%	11.0%	
Grade (WHO2004)			0.17			0.10
Low grade	36 (34.3%)	153 (42.1%)		46.7%	42.0%	
High grade	61 (58.1%)	181 (49.9%)		47.0%	50.7%	
No malignancy	8 (7.6%)	29 (8.0%)		6.3%	7.3%	
Concomitant CIS			0.27			0.02
Yes	22 (21.0%)	41 (11.3%)		12.5%	13.2%	
No	83 (79.0%)	322 (88.7%)		87.5%	86.8%	
LVI			0.02			0.04
Yes	8 (7.6%)	26 (7.2%)		5.4%	6.4%	
No	97 (92.4%)	337 (92.8%)		94.6%	93.6%	
Second TUR			0.07			0.05
Yes	16 (15.2%)	46 (12.7%)		11.4%	13.0%	
No	89 (84.8%)	317 (87.3%)		88.6%	87.0%	
Induction BCG therapy			0.22			0.10
Yes	24 (22.9%)	52 (14.3%)		11.9%	15.4%	
No	81 (77.1%)	311 (85.7%)		88.1%	84.6%	
Maintenance BCG therapy			0.06			0.07
Yes	5 (4.8%)	13 (3.6%)		2.6%	3.8%	
No	100 (95.2%)	350 (96.4%)		97.4%	96.2%	

BCG, Bacillus Calmette–Guérin; BMI, body mass index; BPH, benign prostatic hyperplasia; CIS, carcinoma in situ; DPP4i, dipeptidyl peptidase-4 inhibitor; ECOG-PS, Eastern Cooperative Oncology Group Performance Status; GLP-1, glucagon-like peptide-1; LVI, lymphovascular invasion; MIBC, muscle-invasive bladder cancer; NB, neurogenic bladder; OAB, overactive bladder; TURBT, transurethral resection of bladder tumor; SD, standard deviation; SMD, standardized mean difference; WHO, World Health Organization.

## Data Availability

The original contributions presented in the study are included in the article/supplementary material, further inquiries can be directed to the corresponding author.

## Supplementary Materials

The following supporting information can be downloaded at: https://www.mdpi.com/article/10.3390/diagnostics15141824/s1, Supplementary Table S1. IPTW-adjusted Cox proportional hazards model for fUTI-free survival in DM group. Supplementary Table S2. IPTW-adjusted Cox proportional hazards model for duration of pyuria in DM group.

## Abbreviations

The following abbreviations are used in this manuscript:

BCG	Bacille Calmette–Guérin
CI	confidence interval
CIS	carcinoma in situ
DM	diabetes mellitus
ECOG-PS	Eastern Cooperative Oncology Group-performance status
fUTI	febrile urinary tract infection
HR	hazard ratio
IPTW	inverse probability of treatment weighting
IVRFS	intravesical recurrence-free survival
LVI	lymphovascular invasion
MIBC	muscle invasive bladder cancer
PFS	progression-free survival
SD	standard deviation
SGLT2i	sodium-glucose cotransporter-2 inhibitor
SMD	standardized mean differences
T2DM	type 2 diabetes mellitus
TURBT	transurethral resection of bladder tumor
UTI	urinary tract infection
UTUC	upper urinary tract urothelial carcinoma
