# Supplementary material for: Impact of Sodium-Glucose Cotransporter-2 Inhibitors on Post-Transurethral Resection of Bladder Tumor Infection and Prognosis
Source: Diagnostics (Basel). 2025 Jul 20;15(14):1824. doi: 10.3390/diagnostics15141824 (PMC12293774; doi:10.3390/diagnostics15141824)
Supplement: Supplementary file 1 [file diagnostics-15-01824-s001.zip › diagnostics-3696262-supplementary.pdf]

Supplementary Table S1. IPTW-adjusted Cox proportional hazards model for fUTI-free survival in DM group†							
Variables	Univariate analysis				Multivariate analysis		
	HR	95% CI	*P* value		HR	95% CI	*P* value
Age ≥70 years	1.03	1.00–1.06	0.05		1.97	0.76–5.11	0.17
Sex Female	1.92	0.78–4.76	0.16				
PS 1–3	1.70	0.88–3.24	0.10		1.59	0.72–3.51	0.24
Smoking history	0.89	0.47–1.69	0.73				
BMI ≥25 kg/m^2	0.66	0.33–1.31	0.23				
Urinary catheter placement	2.46	0.39–15.41	0.34				
Heart disease	1.04	0.52–2.06	0.92				
BPH	0.69	0.33–1.44	0.32				
NB	3.50	1.25–9.77	0.02		2.77	0.88–8.67	0.08
OAB	1.35	0.48–3.76	0.57				
History of pelvic radiation therapy	1.72	0.43–6.98	0.45				
eGFR <60mL/min/1.73m^2	0.85	0.47–1.54	0.60				
HbA1c ≥7%	0.97	0.72–1.32	0.86				
Antidiabetic drugs							
Insulin	1.20	0.47–3.06	0.71				
SGLT2i	2.29	1.15–4.59	0.02		2.36	1.22–4.56	0.01
Sulfonylureas	0.30	0.10–0.88	0.03		0.35	0.12–1.03	0.06
Biguanides	0.91	0.45–1.84	0.79				
DPP4i	1.10	0.57–2.12	0.78				
Thiazolidinediones	1.41	0.48–4.13	0.53				
α-glucosidase inhibitors	0.40	0.09–1.78	0.23				
Meglitinides	1.59	0.39–6.35	0.51				
Bacteriuria at TURBT	2.09	1.08–4.03	0.03		1.70	0.53–5.46	0.37
Pyuria at TURBT	1.82	0.95–3.51	0.07		1.25	0.40–3.94	0.70
History of recurrence	0.57	0.25–1.31	0.19				
Tumor size ≥30mm	2.56	1.29–5.10	<0.01		1.47	0.73–2.92	0.28
Multiple tumors	2.42	1.23–4.78	0.01		1.96	1.01–3.79	0.05
pTis tumor	2.04	0.57–7.37	0.27				
pT1 tumor	1.24	0.36–4.25	0.74				
MIBC	3.24	0.96–10.91	0.06		1.47	0.32–6.72	0.62
High grade tumor (WHO2004)	2.20	0.96–5.00	0.06		1.50	0.41–5.46	0.54
Concomitant CIS	0.97	0.38–2.46	0.95				
LVI	4.04	1.89–8.61	<0.001		2.42	0.81–7.24	0.15

BCG, Bacillus Calmette-Guérin; BMI, body mass index; BPH, benign prostatic hyperplasia; CIS, carcinoma in situ; DPP4i, dipeptidyl peptidase-4 inhibitor; ECOG-PS, Eastern Cooperative Oncology Group Performance Status; GLP-1, glucagon-like peptide-1; LVI, lymphovascular invasion; MIBC; muscle-invasive bladder cancer; NB, neurogenic bladder; OAB, overactive bladder; TURBT, transurethral resection of bladder tumor; SD, standard deviation; SMD, standardized mean difference; WHO, world health organization

† Adjusted with second TUR, induction BCG therapy and maintenance BCG therapy

Supplementary Table S2. IPTW-adjusted Cox proportional hazards model for duration of pyuria in DM group†							
Variables	Univariate analysis				Multivariate analysis		
	HR	95% CI	*P* value		HR	95% CI	*P* value
Age ≥70 years	1.00	0.99–1.02	0.78				
Sex Female	0.95	0.68–1.35	0.80				
PS 1–3	0.96	0.72–1.27	0.76				
Smoking history	1.16	0.92–1.45	0.47				
BMI ≥25 kg/m^2	0.99	0.79–1.24	0.93				
Urinary catheter placement	1.10	0.59–2.04	0.77				
Heart disease	1.11	0.87–1.41	0.40				
BPH	0.98	0.80–1.29	0.89				
NB	0.95	0.77–1.75	0.87				
OAB	1.10	0.76–1.59	0.60				
History of pelvic radiation therapy	1.09	0.70–1.69	0.71				
eGFR <60mL/min/1.73m^2	1.41	1.14–1.75	<0.01		1.41	1.14–1.79	<0.01
HbA1c ≥7%	1.00	0.80–1.23	0.80				
Antidiabetic drugs							
Insulin	1.33	0.94–1.89	0.11				
SGLT2i	2.13	1.49–2.94	<0.001		1.52	1.03–2.22	0.04
Sulfonylureas	1.19	0.85–1.54	0.20				
Biguanides	1.10	0.85–1.41	0.48				
DPP4i	0.77	0.61–0.96	0.02		0.74	0.59–0.93	0.01
Thiazolidinediones	0.76	0.54–1.08	0.12				
α -glucosidase inhibitors	0.88	0.59–1.30	0.51				
Meglitinides	0.99	0.59–1.67	0.96				
Bacteriuria at TURBT	1.22	0.98–1.52	0.07		1.18	0.93–1.49	0.16
Pyuria at TURBT	1.10	0.88–1.35	0.39				
History of recurrence	0.90	0.70–1.15	0.38				
Tumor size ≥30mm	1.03	0.79–1.32	0.87				
Multiple tumors	1.10	0.88–1.37	0.42				
pTis tumor	1.52	0.92–2.50	0.11				
pT1 tumor	1.09	0.76–1.56	0.65				
MIBC	0.99	0.64–1.52	0.94				
High grade tumor (WHO2004)	0.91	0.71–1.14	0.37				
Concomitant CIS	1.30	0.89–1.89	0.17				
LVI	0.91	0.63–1.32	0.61				

BCG, Bacillus Calmette-Guérin; BMI, body mass index; BPH, benign prostatic hyperplasia; CIS, carcinoma in situ; DPP4i, dipeptidyl peptidase-4 inhibitor; ECOG-PS, Eastern Cooperative Oncology Group Performance Status; GLP-1, glucagon-like peptide-1; LVI, lymphovascular invasion; MIBC; muscle-invasive bladder cancer; NB, neurogenic bladder; OAB, overactive bladder; TURBT, transurethral resection of bladder tumor; SD, standard deviation; SMD, standardized mean difference; WHO, world health organization

† Adjusted with second TUR, induction BCG therapy and maintenance BCG therapy
